# Mesenchymal Stem Cells Attenuate Cisplatin-Induced Nephrotoxicity in iNOS-Dependent Manner

**DOI:** 10.1155/2017/1315378

**Published:** 2017-07-30

**Authors:** Bojana Simovic Markovic, Marina Gazdic, Aleksandar Arsenijevic, Nemanja Jovicic, Jovana Jeremic, Valentin Djonov, Nebojsa Arsenijevic, Miodrag L. Lukic, Vladislav Volarevic

**Affiliations:** ^1^Center for Molecular Medicine and Stem Cell Research, Faculty of Medical Sciences, University of Kragujevac, 34000 Kragujevac, Serbia; ^2^Department of Genetics, Faculty of Medical Sciences, University of Kragujevac, 34000 Kragujevac, Serbia; ^3^Department of Histology and Embryology, Faculty of Medical Sciences, University of Kragujevac, 34000 Kragujevac, Serbia; ^4^Department of Pharmacy, Faculty of Medical Sciences, University of Kragujevac, 34000 Kragujevac, Serbia; ^5^Institute of Anatomy, University of Bern, 3000 Bern 9, Switzerland

## Abstract

Mesenchymal stem cells (MSCs) are, due to their immunomodulatory characteristics, utilized in therapy of immune-mediated diseases. We used murine model of cisplatin nephrotoxicity to explore the effects of MSCs on immune cells involved in the pathogenesis of this disease. Intraperitoneal application of MSCs significantly attenuated cisplatin nephrotoxicity, decreased inflammatory cytokines TNF-*α* and IL-17, and increased anti-inflammatory IL-10, IL-6, nitric oxide (NO), and kynurenine in sera of cisplatin-treated mice. MSC treatment significantly attenuated influx of leukocytes, macrophages, dendritic cells (DCs), neutrophils, CD4+ T helper (Th), and CD8+ cytotoxic T lymphocytes (CTLs) in damaged kidneys and attenuated the capacity of renal-infiltrated DCs, CD4+ Th, and CD8+ CTLs to produce TNF-*α* and IL-17. Similar effects were observed after intraperitoneal injection of MSC-conditioned medium (MSC-CM) indicating that MSCs exert their beneficial effects in paracrine manner. Inhibition of inducible nitric oxide synthase (iNOS) in MSC-CM resulted with increased number of TNF-*α*-producing DCs and IL-17-producing CTLs, decreased number of IL-10-producing tolerogenic DCs and regulatory CD4+FoxP3+ T cells, and completely diminished renoprotective effects of MSC-CM. In conclusion, MSCs, in iNOS-dependent manner, attenuated inflammation in cisplatin nephrotoxicity by reducing the influx and capacity of immune cells, particularly DCs and T lymphocytes, to produce inflammatory cytokines.

## 1. Introduction

Acute renal injury, characterized by tubular cell damage and kidney dysfunction, may be developed as a consequence of drug-induced toxicity [1]. As one of the most effective chemotherapeutics, cisplatin has been used for the therapy of a broad range of solid tumors including lung, ovarian, bladder, gastric, and testicular cancers [2]. However, clinical application of cisplatin is limited because of nephrotoxicity that, as a serious side effect, occurs in nearly 30% of cisplatin-treated patients [3]. Cisplatin, which could be metabolized to a potent nephrotoxin-reactive thiol, selectively accumulates in proximal tubular cells to five times higher degree of the serum concentration and damages proximal tubular epithelial cells, contributing to nephrotoxicity [3]. Acute cisplatin-induced nephrotoxicity is associated with a robust inflammatory response followed by infiltration of immune cells that promotes further progression of renal tissue damage leading to the development of renal failure [4, 5].

In many cisplatin-treated patients, kidney injury, manifested by increased serum creatinine and blood urea nitrogen (BUN) levels and decreased renal blood flow, hypomagnesemia, hypocalcemia, and proteinuria [3], is irreversible, requiring substitution, reduction, or discontinuation of cisplatin treatment. Since currently there is no compatible and convenient chemotherapeutic agent with similar potent, anticancer efficacy as cisplatin, the clinical use of cisplatin cannot be abandoned. Accordingly, an urgent demand exists for researchers to develop new adjuvant therapy for attenuation of cisplatin-induced nephrotoxicity and inflammation.

Mesenchymal stem cells (MSCs) are adult, self-renewable, multipotent cells which are, due to their differentiation and immunomodulatory characteristics, used in preclinical and clinical studies of degenerative and immune-mediated diseases [6–8].

Recently, MSC-based therapeutic approach for attenuation of cisplatin-induced nephrotoxicity has been extensively investigated, and several possible mechanisms were proposed. As demonstrated by Ashour et al. [9], renoprotective effects of intraperitoneally injected MSCs were based on MSC-mediated reduction of oxidative stress. Significantly decreased renal tissue malondialdehyde, increased reduced glutathione level, and superoxide dismutase activity were noticed in cisplatin-treated rats that received MSCs [9].

Additionally, Kim et al. [10] and Yao et al. [11] demonstrated that MSCs markedly improved cisplatin-induced renal failure by suppressing apoptosis in paracrine and p53-dependent manner. Alleviated kidney injury was accompanied by decreased expression of cyclooxygenase COX-2 and tumor necrosis factor alpha (TNF-*α*), a key mediator in the inflammatory response triggered by cisplatin, indicating that MSC attenuated cisplatin-induced nephrotoxicity by modulating kidney inflammation. In line with these findings, Park et al. [12] recently showed that early but not late treatment with MSCs attenuates cisplatin-induced nephrotoxicity and modulates inflammation in the injured kidneys.

Although data presented in these studies [9–12] clearly demonstrated that MSCs were able to protect the kidney from the cisplatin-induced toxicity, the effects of MSC on immune cell-mediated mechanisms involved in induction and progression of cisplatin-caused kidney inflammation are still unknown. The aim of our study was to investigate the cellular mechanisms underlying the protective effects of MSCs on kidney function in order to suggest new pathways that can be used for the modulation of MSC-dependent protection of cisplatin-induced nephrotoxicity.

Herewith, we show that single intraperitoneal injection of MSCs and MSC-conditioned medium (MSC-CM) diminished influx of immune cells (dendritic cells (DCs), macrophages, neutrophils, effector CD4+ T helper, and cytotoxic CD8+ CTL lymphocytes) into the cisplatin-injured kidneys and attenuated their capacity to produce nephrotoxic and inflammatory cytokines (TNF-*α* and interleukin- (IL-) 17) in inducible nitric oxide synthase- (iNOS-) dependent manner.

## 2. Materials and Methods

### 2.1. Cells

Mouse bone marrow-derived MSCs were purchased from Gibco (catalog number S1502-100). The cells were cultured in complete Dulbecco's Modified Eagle Medium (DMEM) containing 10% heat-inactivated fetal bovine serum (FBS), 100 IU/mL penicillin G, and 100 *μ*g/mL streptomycin (Sigma-Aldrich, Munich, Germany), at 37°C in a 5% CO_2_ incubator. MSCs in passage 4 were used throughout the experiments.

### 2.2. Generation of MSC-Conditioned Medium (MSC-CM)

MSCs were seeded at a density of 10,000 cells/cm^2^. In order to collect the MSC-CM, MSCs were first cultured in serum-containing complete medium and incubated at 37°C in a humid atmosphere with 5% CO_2_. At 80% confluence, the cells were washed twice with 1x phosphate-buffered saline (PBS, Invitrogen), and the medium was then changed to serum-free medium. After 48 h, the medium was collected, centrifuged at 13000 ×g at 4°C for 10 min, and stored at −80°C until used [13].

### 2.3. Pharmacological Inhibition of iNOS and Indoleamine 2,3-Dioxygenase (IDO)

To block iNOS activity, mMSCs or hMSCs were cultured for 48 h in the presence of 1 mM of an iNOS inhibitor, L-N^G^-monomethyl arginine citrate (L-NMMA, Sigma-Aldrich, St. Louis, MO) [14].

MSCs were cultured for 48 h in culture medium containing 1 mM 1-methyltryptophan, (1-MT, Sigma-Aldrich, St. Louis, MO), an inhibitor of IDO enzymatic activity [15].

### 2.4. In Vitro Activation of MSCs

For in vitro activation, MSCs were cultured 48 h in the presence of 10 ng/mL recombinant mouse TNF-*α* (Ebioscience, San Diego, USA). IDO activity in supernatants of TNF-*α*-activated MSCs was determined by spectrophotometric measuring of kynurenine (described under Section 2.11) while expression of iNOS and IDO was determined by real-time RT-PCR, as described under Section 2.12.

### 2.5. Animals

6–8-week-old male BALB/c mice were randomly divided in control and experimental groups (*n* = 10 mice/group). All animals received human care, and all experiments were approved by and conducted in accordance with the Guidelines of the Animal Ethics Committee of the Faculty of Medical Sciences of the University of Kragujevac, Serbia. Mice were housed in a temperature-controlled environment with a 12 hour light-dark cycle and were administered with standard laboratory chow and water ad libitum.

### 2.6. Induction of Cisplatin Nephrotoxicity and Application of MSCs and MSC-CM

Cisplatin nephrotoxicity was induced by intraperitoneal injection of cisplatin (16 mg/kg body weight) [16]. One hour after the injection of cisplatin, MSC-treated mice intraperitoneally received 5 × 10^5^ MSCs and resuspended in 200 *μ*L of saline, while MSC-CM-treated mice intraperitoneally received 200 *μ*L of MSC-CM. Mice were randomized to receive cisplatin only, cisplatin and MSCs, cisplatin and MSC-CM, MSCs, MSC-CM, or saline only (control mice). After mouse euthanasia (72 h after cisplatin treatment), both the kidneys were excised and blood samples were drawn from the inferior vena cava, as previously described [16].

### 2.7. Determination of BUN and Creatinine Levels

Serum levels of BUN and creatinine were determined to assess the renal function. After blood collection, serum levels of these toxicity markers were measured immediately using assay kits and blood chemistry analyzer, as described [17].

### 2.8. Histopathological Analysis

The isolated kidneys were fixed in 10% formalin and embedded in paraffin, and consecutive 5 *μ*m tissue sections were mounted on slides. Sections were stained with Hematoxylin and Eosin (H&E) and examined under low-power (100x) light microscopy- (Zeiss Axioskop 40, Jena, Germany) equipped digital camera. Histological sections were scored using a semiquantitative scale designed to assess acute kidney injury-associated tubular injury (tubular epithelial cell loss, necrosis, tubular epithelial simplification, intratubular debris, and casts) by a pathologist unaware of the experimental groups (using >5 random fields/section, 4-5 mice/group). Tubule injury scores (ranging between 0 and 4) were based on the percentage of tubules affected as follows: 0 ≤ 10%, 1 = 10–25%, 2 = 26–50%, 3 = 51–75%, and 4 ≥ 75%, as previously described [18].

Periodic acid-Schiff (PAS) staining was performed on paraffin-embedded kidney tissue sections using PAS Kit (Sigma-Aldrich, St. Louis, MO) according to the manufacturer's protocol.

### 2.9. Isolation of Renal-Infiltrated Immune Cells

The kidneys were washed with sterile phosphate-buffered saline (PBS) and placed in Petri dishes with DMEM supplemented with 10% FBS. The kidneys were cut into small pieces (1-2 mm in dimension) using a regular metal shaping blade and placed into the collagenase solution for 30–45 min in the incubator at 37°C. The cells were filtered through a 70 *μ*m nylon cell strainer into a clean 50 mL conical tube. Then, cells were pelleted by centrifuging 10 min at 400 ×g, at 4°C. Pellet was resuspended in 4 mL of 40% Percoll solution and gently overlaid onto 4 mL of 80% Percoll solution. Slight whitish translucent layers of cells were collected from the interface of the two Percoll phases after centrifugation at 1500 ×g for 30 minutes, at room temperature. These cells were then collected and pelleted by centrifuging 10 min at 400 ×g, at 4°C. Pellet was resuspended in 1 mL of DMEM, and the total number of cells was determined by using trypan blue exclusion on a hemocytometer [19].

### 2.10. Flow Cytometry Analysis and Intracellular Staining of Renal-Infiltrated Immune Cells

Renal-infiltrated immune cells were screened for various cell surface and intracellular markers with flow cytometry. Briefly, 1 × 10^6^ cells were incubated with anti-mouse CD45, F4/80, CD4, CD8, CD11c, CD11b, Ly6G, and monoclonal antibodies conjugated with fluorescein isothiocyanate (FITC), phycoerythrin (PE), peridinin chlorophyll protein (PerCP), or allophycocyanin (APC) (all from BD Biosciences, San Jose, CA, USA) following the manufacturer's instructions. Immune cells derived from the kidneys were concomitantly stained for the intracellular content of TNF-*α*, IL-10, IL-17, and forkhead box P3 (FoxP3) by using the fixation/permeabilization kit and anti-mouse monoclonal antibodies conjugated with fluorescein isothiocyanate (FITC), phycoerythrin (PE), peridinin chlorophyll protein (PerCP), and allophycocyanin (APC) (BD Bioscience). For intracellular cytokine staining, cells were stimulated with 50 ng/mL PMA and 500 ng/mL ionomycin for 5 h, and GolgiStop (BD Biosciences) was added. Cells were fixed in Cytofix/Cytoperm, permeated with 0.1% saponin, and stained with fluorescent Abs. Flow cytometric analysis was conducted on a BD Biosciences FACSCalibur and analyzed by using the Flowing Software analysis program.

### 2.11. Measurement of Cytokines and Growth Factors

Levels of TNF-*α*, IL-17, IL-10, and IL-6 in the mouse serum were measured using ELISA kits specific for the mouse cytokines (R&D Systems, Minneapolis, MN, USA) according to the manufacturer's instructions.

Serum concentrations of nitric oxide (NO) were measured by Griess reagent while IDO activity in serum and supernatants of TNF-*α*-stimulated MSCs was determined by spectrophotometric measuring of kynurenine since IDO catalyzes the metabolism of tryptophan in the kynurenine [20].

### 2.12. Expression of Genes in Cisplatin-Injured Kidneys and in TNF-*α*-Activated MSCs

Total RNA was extracted from frozen mouse kidneys (for determination of IL-6 and TNF-*α*) or from cultured MSCs (for determination of NO and IDO) using TRIzol (Invitrogen, Carlsbad, CA) according to the manufacturer's instructions. Total RNA (2 *μ*g) was reverse transcribed to cDNA using High-Capacity cDNA Reverse Transcription Kit (Applied Biosystems, Foster City, California, USA). qRT-PCR was performed using Power SYBR MasterMix (Applied Biosystems) and miRNA-specific primers for IL-6, TNF-*α*, NO, IDO, and *β*-actin as a housekeeping gene. qPCR reactions were initiated with a 10-minute incubation time at 95°C followed by 40 cycles of 95°C for 15 seconds and 60°C for 60 seconds in a Mastercycler ep realplex (Eppendorf, Hamburg, Germany). Relative expression of genes was calculated according to the formula 2^−(C_t_ − C_tactin_)^, where *C*_t_ is the cycle threshold of the gene of interest and *C*_tactin_ is the cycle threshold value of the housekeeping gene (*β*-actin) [21].

### 2.13. Statistical Analysis

The results were analyzed using Student's *t*-test. All data in this study were expressed as the mean ± standard error of the mean (SEM). Values of *p* < 0.05 were considered as statistically significant.

## 3. Results

### 3.1. Intraperitoneal Application of MSCs Significantly Attenuates Cisplatin-Induced Acute Kidney Injury

Cisplatin caused significant renal dysfunction as determined by biochemical analysis and histological examination.

**Figure 1:**
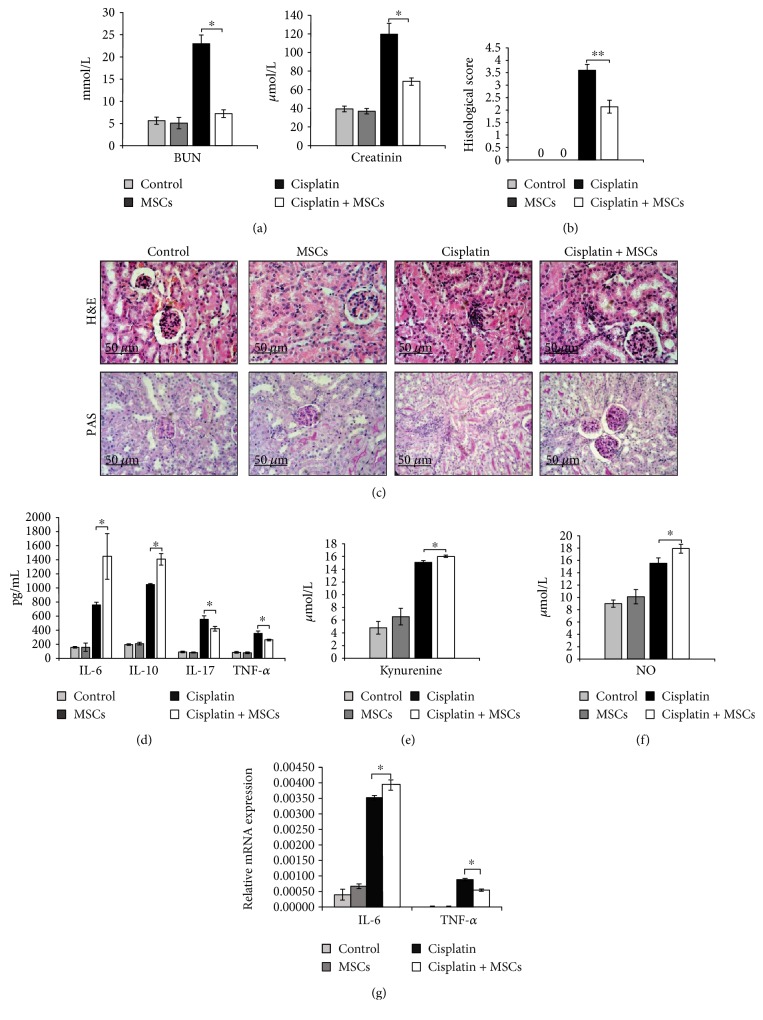
MSCs attenuate cisplatin-induced acute kidney injury. (a) Blood urea nitrogen (BUN) and plasma creatinine levels are evaluated. (b) Histological scores (ranging between 0 and 4) were determinated and calculated on the percentage of tubules affected (0 ≤ 10%, 1 = 10–25%, 2 = 26–50%, 3 = 51–75%, and 4 ≥ 75%). (c) Representative H&E- and PAS-stained mouse kidney. H&E staining images of kidney tissue samples are shown at the same magnifications (×200). Concentration of (d) cytokines, (e) kynurenine, and (f) NO in mice sera. (g) IL-6 and TNF-*α* gene expression in mouse kidneys. Values are mean ± SEM; *n* = 10 mice/group. ^∗^*p* < 0.05, ^∗∗^*p* < 0.001.

As shown in Figure 1(a), cisplatin administration resulted with 4-fold increase in BUN and creatinine when compared to control mice, indicating the induction of severe nephrotoxicity. Single, intraperitoneal injection of MSCs did not alter serum levels of BUN and creatinine in cisplatin-untreated mice. However, MSCs significantly downregulated serum levels of both BUN (*p* < 0.05) and creatinine (*p* < 0.05) in cisplatin-treated animals suggesting beneficent effects of MSCs in the treatment of cisplatin-induced nephrotoxicity.

As shown in Figure 1(c), the kidneys obtained from control and MSC-only treated animals had normal histology. Partial tubular cell necrosis with citoplasmatic vacuolar transformation of the tubular epithelium due to hydropic degeneration and mild interstitial edema with discrete focal monocyte infiltration was noticed in cisplatin-treated mice. On the contrary, cisplatin + MSC-treated mice showed significant reduction in renal injury followed by reduced infiltration of inflammatory cells (Figure 1(c)). The histological scores also showed increased tubular injury score after cisplatin treatment, which was significantly reversed by MSCs (Figure 1(b)).

In accordance with the biochemical and histological analysis, MSCs did not affect serum levels of cytokines in cisplatin-untreated mice indicating that the differences in their concentration, between cisplatin + MSC-treated and cisplatin-treated mice (Figure 1(d)), are a consequence of MSC-mediated suppression of immune cells that produce these mediators. The concentrations of nephrotoxic and inflammatory cytokines TNF-*α* (*p* < 0.05) and IL-17 (*p* < 0.05) were significantly lower while concentrations of anti-inflammatory IL-10 (*p* < 0.01) and IL-6 (*p* < 0.05) were significantly higher in sera of cisplatin-treated mice that received MSCs (Figure 1(d)). In line with these findings, the expression of TNF-*α* was significantly lower (*p* < 0.05) while expression of IL-6 was significantly higher (*p* < 0.05) in the kidneys of cisplatin + MSC-treated mice when compared to animals that received only cisplatin (Figure 1(g)). Immunosuppressive kynurenine (*p* < 0.05, Figure 1(e)) and NO (*p* < 0.05, Figure 1(f)) were also elevated in the serum of cisplatin + MSC-treated mice suggesting that the production of IDO and NO by MSCs may be important for their beneficent effects.

### 3.2. Influx of Immune Cells and Their Capacity to Produce Nephrotoxic and Inflammatory Cytokines Have Been Significantly Attenuated by MSCs

**Figure 2:**
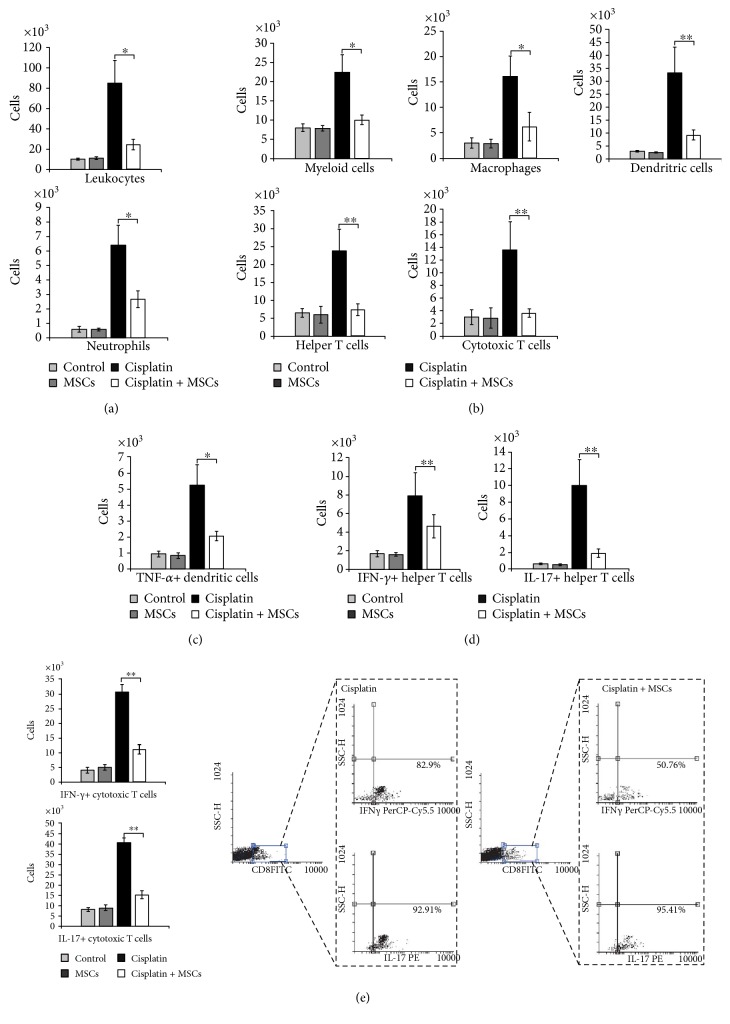
MSCs significantly attenuate influx of immune cells and their capacity to produce nephrotoxic and inflammatory cytokines. Total number of (a) CD45+ leukocytes, (b) CD45+CD11b+ myeloid cells, CD45+F4/80+ macrophages, CD45+CD11c+ dendritic cells, CD45+CD11b+Ly6G+ neutrophils, CD45+CD4+ T helper cells, CD45+CD8+ cytotoxic T cells, (c) TNF-*α*+CD11c+ dendritic cells, (d) IFN-*γ*+CD4+ T helper cells, and IL-17+CD4+ T helper cells in cisplatin- and cisplatin + MSC-treated mice. (e) Total number and representative flow cytometry dot plots of IFN-*γ*- and IL-17-producing cytotoxic CD8+ T cells. Data presented as mean ± SEM; *n* = 10 mice/group. ^∗^*p* < 0.05, ^∗∗^*p* < 0.01.

To assess the role of MSCs for inflammatory cell accumulation in the kidneys after cisplatin injection, different populations of renal-infiltrated immune cells were analyzed by flow cytometry. MSCs did not alter the total number of renal-infiltrated immune cells in cisplatin-untreated animals. Nevertheless, in cisplatin-treated mice, influx of immune cells and their capacity to produce nephrotoxic and inflammatory cytokines have been significantly attenuated by MSCs. As shown in Figure 2(a), 72 hours after cisplatin injection, accumulation of CD45+ leukocytes was much less pronounced (*p* < 0.05) in the kidneys from cisplatin + MSC-treated mice compared to cisplatin-only-treated animals.

Cellular make-up of the kidneys (Figure 2(b)) showed that MSC treatment significantly attenuated influx of CD45+CD11b+ myeloid cells (*p* < 0.05), CD45+F4/80+ macrophages (*p* < 0.05), CD45+CD11c+DCs (*p* < 0.01), CD45+CD11b+Ly6G+ neutrophils (*p* < 0.05), CD45+CD4+ helper T cells (*p* < 0.01), and CD45+CD8+ cytotoxic T cells (CTLs) (*p* < 0.01).

Moreover, MSCs attenuate the capacity of DCs, CD4+T helper, and CD8+ CTLs to produce inflammatory cytokines in cisplatin-injured kidneys. Intracellular staining revealed significantly decreased number of TNF-*α*-producing DCs (Figure 2(c), *p* < 0.05), IFN-*γ*- and IL-17-producing CD4+ T cells (Figure 2(d), *p* < 0.01), and IFN-*γ*- and IL-17-producing CD8+ CTLs (Figure 2(e), *p* < 0.01) in the kidneys of cisplatin-injured mice that received MSCs when compared to the cisplatin-only-treated animals.

### 3.3. MSCs Attenuate Cisplatin-Induced Nephrotoxicity in Paracrine Manner

To investigate whether soluble factors were responsible for the MSC-mediated attenuation of cisplatin-induced nephrotoxicity, cisplatin-treated mice intraperitoneally received MSC-CM.

**Figure 3:**
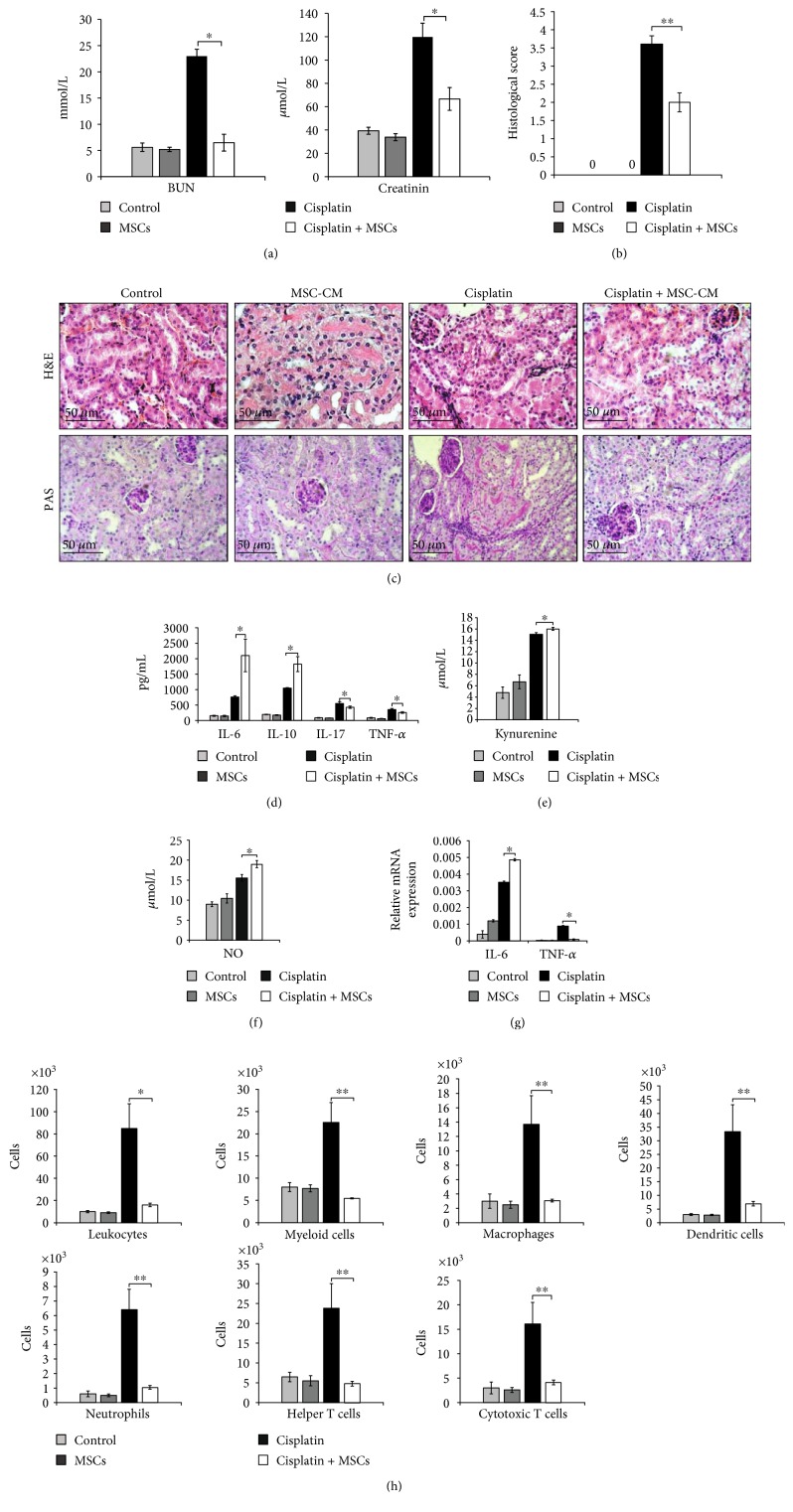
MSCs reduce cisplatin-induced nephrotoxicity via soluble factors. (a) Mice were euthanized 72 h after cisplatin administration, and blood urea nitrogen (BUN) and plasma creatinine levels are measured. (b) Histological examination was performed with H&E staining. (c) H&E and PAS staining images of representative kidney tissues are shown at the same magnifications (200x). Concentration of (d) cytokines, (e) kynurenine, and (f) NO in mouse serum. (g) Expression of IL-6 and TNF-*α* genes in mouse kidneys. (h) Total number of renal-infiltrated CD45+ leukocytes, CD45+CD11b+ myeloid cells, CD45+F4/80+ macrophages, CD45+CD11c+ dendritic cells, CD45+CD11b+Ly6G+ neutrophils, CD45+CD4+ T helper cells, and CD45+CD8+ cytotoxic T cells. Data presented as mean ± SEM; *n* = 10 mice/group. ^∗^*p* < 0.05, ^∗∗^*p* < 0.01.

Biochemical analysis showed that MSC-CM did not alter serum levels of BUN and creatinine in cisplatin-untreated mice but managed to markedly decrease both BUN and creatinine in cisplatin-treated animals (Figure 3(a)).

Histological analysis revealed reduced necrosis, vacuolization, and desquamation of epithelial cells in the renal tubules of cisplatin + MSC-CM-treated mice when compared to the animals that received only cisplatin (Figure 3(c)). Histological score confirmed significant reduction of acute renal injury in cisplatin-treated mice that received MSC-CM (Figure 3(b)).

As shown in Figures 3(d), 3(e), and 3(f), MSC-CM treatment significantly downregulated serum levels of nephrotoxic and inflammatory TNF-*α* (*p* < 0.05) and IL-17 (*p* < 0.05) and increased serum levels of immunosuppressive IL-10 (*p* < 0.05), IL-6 (*p* < 0.05), kynurenine (*p* < 0.05), and NO (*p* < 0.05) in cisplatin-treated mice. Accordingly, MSC-CM significantly downregulated expression of TNF-*α* (*p* < 0.05) and increased expression of IL-6 (*p* < 0.05) in the kidneys of cisplatin-treated animals (Figure 3(g)).

### 3.4. MSC-CM Decreases Inflammatory Cell Accumulation in the Kidneys of Cisplatin-Treated Mice

**Figure 4:**
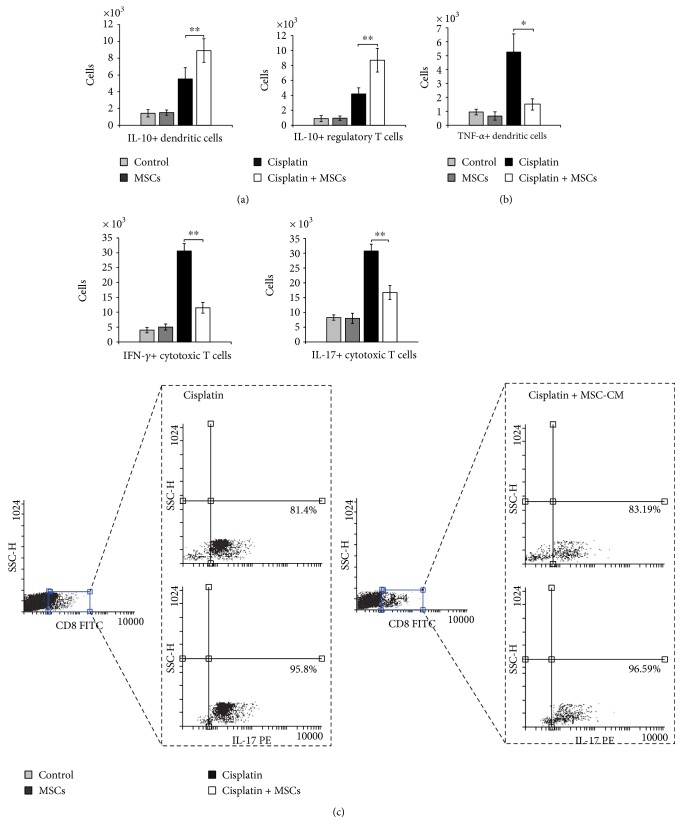
MSC-CM reduces influx of inflammatory DCs and CTLs in cisplatin-induced acute kidney injury and alters their cytokine profile. Total number of (a) IL-10-producing CD45+CD11c+ DCs, CD4+CD25+FoxP3+ T regulatory cells, (b) TNF-*α*+CD45+CD11c+ DCs, (c) IFN-*γ*- and IL-17-producing CD8+ CTL cells that infiltrated kidneys of the control and experimental animals. Representative flow cytometry dot plots are shown. Values are mean ± SEM; *n* = 10 mice/group. ^∗^*p* < 0.05, ^∗∗^*p* < 0.001.

Application of MSC-CM did not affect influx of renal-infiltrated immune cells in cisplatin-untreated animals (Figures 3(h) and 4(a), 4(b), and 4(c)). Nevertheless, similar as it was observed after injection of MSCs, MSC-CM managed to significantly reduce the presence of CD45+ leukocytes (*p* < 0.05), CD45+CD11b+ myeloid cells (*p* < 0.01), CD45+F4/80+ macrophages (*p* < 0.01), CD45+CD11c+ DCs (*p* < 0.01), CD45+CD11b+Ly6G+ neutrophils (*p* < 0.01), CD45+CD4+ helper T cells (*p* < 0.01), and CD45+CD8+ CTLs (*p* < 0.01) in the kidneys of cisplatin-injured mice (Figure 3(g)).

As determined by intracellular staining, in comparison to cisplatin-only-treated mice, MSC-CM treatment significantly increased the total number of immunosuppressive IL-10-producing DCs (*p* < 0.01, Figure 4(a), left panel) and regulatory T cells (*p* < 0.01, Figure 4(a), right panel) and attenuated the total number of inflammatory TNF-*α*-producing DCs (*p* < 0.05, Figure 4(b)), IFN-*γ*- and IL-17-producing CD8+ cytotoxic T cells (*p* < 0.01, Figure 4(c)).

### 3.5. The Capacity of MSC-CM to Protect from Cisplatin-Induced Acute Kidney Injury Is Completely Abrogated by iNOS Inhibitor

Various mediators are proposed to be responsible for the immunosuppressive effects of MSCs, including NO, IDO, TGF-*β*, HGF, PGE_2_, and IL-10 [22–24]. IDO plays a key role in immunomodulation mediated by human MSCs while murine MSCs mainly use iNOS-dependent suppression of immune response [25]. Accordingly, we investigated the effects of iNOS or IDO inhibition on MSC-CM-dependent attenuation of cisplatin-induced nephrotoxicity.

**Figure 5:**
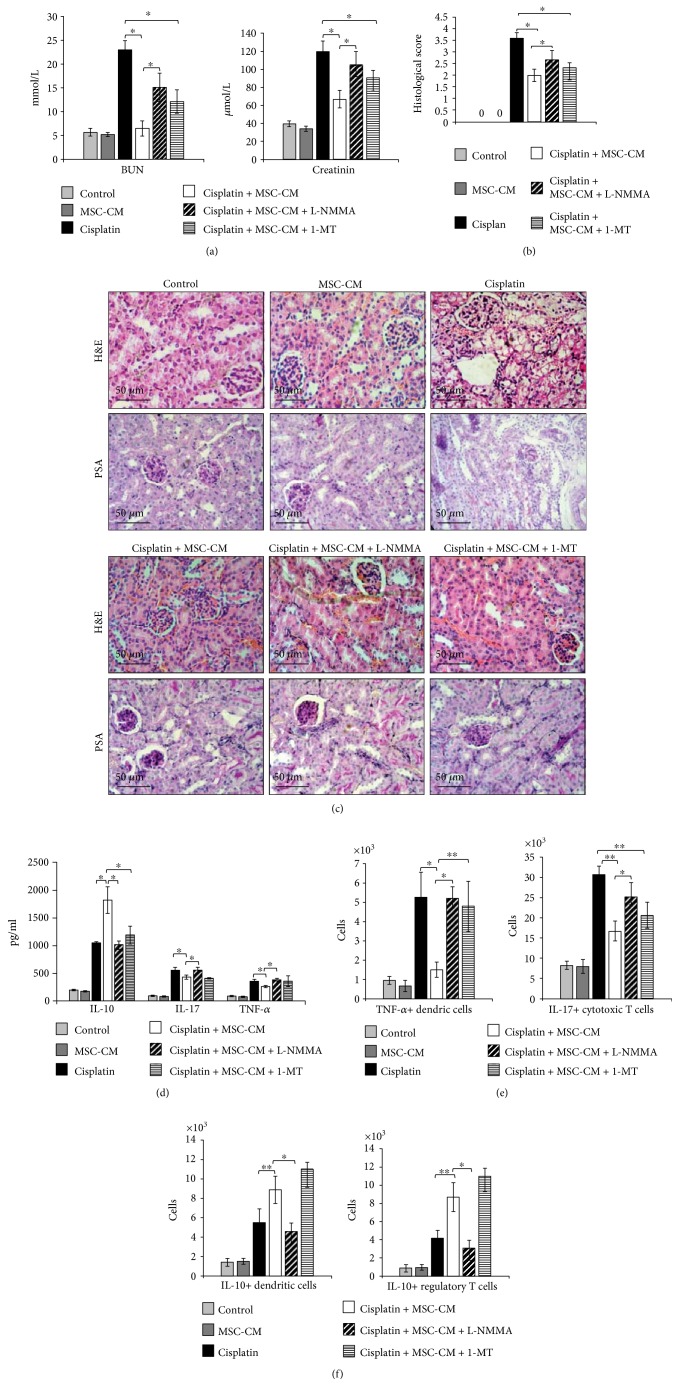
MSCs attenuate cisplatin-induced acute kidney injury in iNOS-dependent manner. (a) Serum levels of BUN and creatinine. (b) Histological scores. (c) Representative H&E and PAS-stained mouse kidney (magnifications ×200). (d) Serum levels of cytokines. Total numbers of (e) TNF-*α*-producing CD45+CD11c+ dendritic cells and IL-17-producing CD8+ cytotoxic T cells, (f) IL-10-producing CD45+CD11c+ DCs and CD4+CD25+FoxP3+ T regulatory cells. Values are mean ± SEM; *n* = 10 mice/group. ^∗^*p* < 0.05, ^∗∗^*p* < 0.001.

As it is shown in Figure 5, blockade of iNOS by L-NMMA almost completely diminished the renoprotective effects of MSC-CM as determined by increased serum levels of BUN and creatinine (*p* < 0.05, Figure 5(a)) and histological analysis (Figures 5(b) and 5(c)).

Almost normal morphology with well-preserved brush border membranes and no loss of tubular epithelial cells was noticed in cisplatin +MSC-CM-treated animals. On the contrary, cisplatin + MSC-CM + L-NMMA-treated kidneys exhibited severe histological changes which included tubular necrosis and dilation (Figure 5(c)). These findings were confirmed by histological scores (Figure 5(b)).

Similar as it was noticed by biochemical and histological analysis, blockade of iNOS resulted with elevated serum levels of TNF-*α* (*p* < 0.05, Figure 5(d)) and decreased serum concentration of immunosuppressive IL-10 (*p* < 0.01, Figure 5(d)) in the kidneys of cisplatin + MSC-CM + L-NMMA-treated mice. Significantly higher number of inflammatory TNF-*α*-producing DCs (*p* < 0.05, Figure 5(e), left panel), followed with increased number of IL-17-producing CTLs (*p* < 0.05, Figure 5(e), right panel), was noticed in the kidneys of cisplatin + MSC-CM + L-NMMA-treated mice when compared to cisplatin + MSC-CM-treated animals (Figure 4(f)). Interestingly, iNOS inhibition also attenuates the capacity of MSC-CM to affect influx of regulatory cells in the injured kidneys. There was significantly lower number of tolerogenic IL-10-producing DCs (*p* < 0.05, Figure 5(f), left panel) and IL-10-producing regulatory T cells (*p* < 0.05, Figure 5(f), right panel) in the kidneys of cisplatin + MSC-CM + L-NMMA-treated mice when compared to cisplatin + MSC-CM-treated animals.

Biochemical and histological analyses indicate that, in contrast to L-NMMA, IDO inhibitor (1-MT) did not manage to completely abrogate renoprotective effects of MSC-CM (Figures 5(a), 5(b), and 5(c)). Significant difference was not observed for serum levels of creatinine and BUN (Figure 5(a)) and histological scores (Figure 5(b)) between cisplatin + MSC-CM- and cisplatin + MSC-CM + 1-MT-treated animals.

In accordance with results obtained by biochemical and histological analyses, cellular make-up of cisplatin-injured kidneys revealed that 1-MT only affected MSC-CM-mediated suppression of TNF-*α* production in DCs (Figure 5(e)) and did not significantly alter MSC-CM-dependent modulation of CTLs and regulatory cells (Figure 5(f)).

### 3.6. iNOS Is Important for Activation of IDO in TNF-*α*-Stimulated MSCs

**Figure 6:**
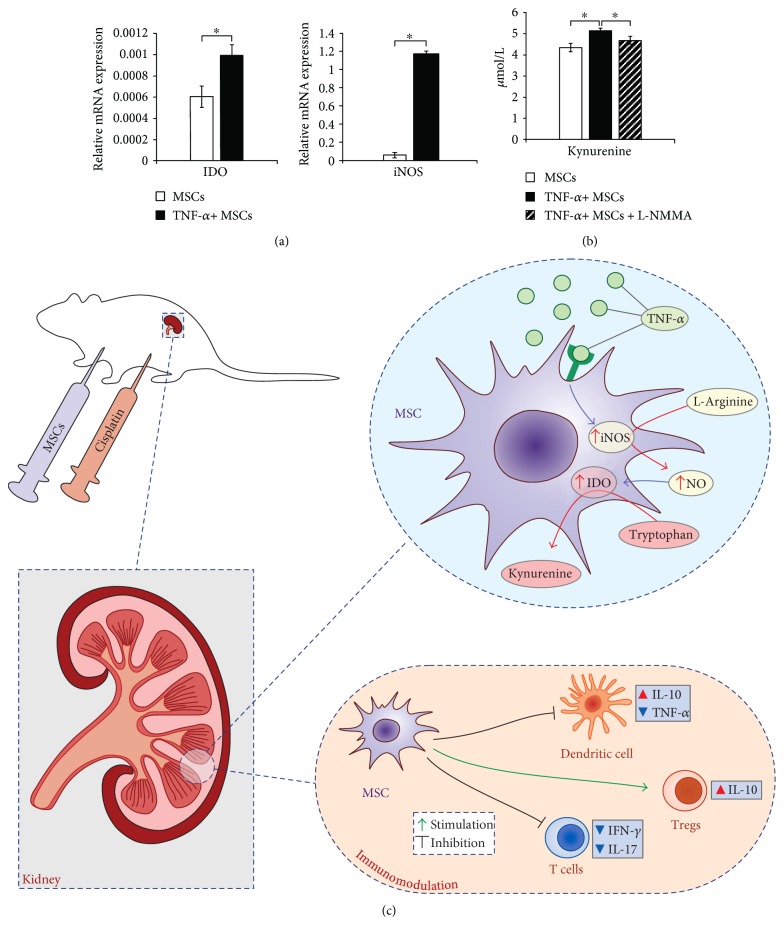
MSC-derived NO is important for activation of IDO in TNF-*α*-stimulated MSCs. (a) Expression of IDO and iNOS in nonstimulated and TNF-*α*-stimulated MSCs. (b) Concentration of kynurenine in supernatants of nonstimulated MSCs, TNF-*α*-stimulated MSCs, and TNF-*α*-stimulated MSCs cultured in the presence of L-NMMA. ^∗^*p* < 0.05. (c) Proposed mechanism of MSC-based immunomodulation of cisplatin-induced nephrotoxicity: After cisplatin-induced kidney injury, MSCs migrate in the kidneys as a response to the inflammatory cytokines and chemokines. Under inflammatory conditions, TNF-*α* provokes MSCs to express iNOS and to produce NO which, in turn, increases IDO activity and augment MSC-based immunomodulation resulting with attenuated number of inflammatory TNF-*α*-producing DCs and IFN-*γ*- and IL-17-producing T cells and increased number of immunosuppressive IL-10-producing DCs and regulatory T cells in injured kidneys.

Nonstimulated MSCs express both iNOS and IDO but their expression significantly increased after activation of MSCs by TNF-*α* (Figure 6(a)). In order to evaluate the interplay between MSC-derived NO and IDO, concentration of kynurenine was measured in supernatants of TNF-*α*-activated MSCs that were cultured with or without iNOS inhibitor, L-NMMA. As shown in Figure 6(b), L-NMMA significantly attenuated the concentration of kynurenine in TNF-*α*-primed MSCs indicating the importance of iNOS for IDO activity in TNF-*α*-stimulated MSCs.

## 4. Discussion

Here, we provide the evidence that intraperitoneal application of MSCs and MSC-CM attenuates cisplatin-induced nephrotoxicity by suppressing infiltration and activation of immune cells in iNOS-dependent manner.

Cisplatin-induced renal injury is followed by increased release of inflammatory TNF-*α*. As a response to these inflammatory cytokines, endothelial cells in injured kidneys increase expression of selectins and chemokines which are involved in leukocyte trafficking, resulting with massive influx of inflammatory cells in injured kidneys [26]. MSCs interact with endothelial cells and, by producing IL-6, downregulate expression of adhesion molecules on endothelial cells, reducing recruitment of leukocytes into the damaged kidneys [27]. Accordingly, decreased expression of TNF-*α* in the kidneys, attenuated serum levels of TNF-*α*, and increased expression of IL-6 in renal tissue accompanied with elevated serum concentration of IL-6, noticed in cisplatin + MSC-treated mice (Figures 1(d) and 1(g)), were accompanied with reduced infiltration of leukocytes in injured kidneys of these animals (Figure 2(a)).

Among renal-infiltrated immune cells, MSC treatment significantly attenuates influx of neutrophils, DCs, macrophages, and CD4+ helper and CD8+ CTLs (Figure 2(b)) in the kidneys of cisplatin-injured mice.

Neutrophils are reported to infiltrate and exacerbate cisplatin-induced acute kidney injury 24 to 48 h after cisplatin administration. Although the extent of neutrophil infiltration coincided with the severity of acute kidney injury and renal dysfunction, their depletion had no impact on the extent of cisplatin-induced nephrotoxicity [27]. As recently demonstrated by Tadagavadi et al. [27], cisplatin-mediated acute kidney injury is not mediated by neutrophils, while DCs play the most important role in stimulation or suppression of inflammation in cisplatin-induced renal failure. Renal DCs are immune sentinels with the ability to induce immunity or tolerance in acute kidney injury. IL-10, mainly produced by renal DCs, attenuates cisplatin nephrotoxicity and protects from cisplatin-mediated acute kidney injury [28]. Additionally, depletion of IL-10-producing and tolerogenic renal-infiltrated DCs results with aggravation of cisplatin-induced nephrotoxicity [28, 29].

It is well known that MSCs may suppress maturation of DCs in IL-10-dependent manner, promoting generation of their tolerogenic, immunosuppressive phenotype [30]. In inflammatory microenvironment, DCs exposed to TNF-alpha (produced by inflammatory cells) and IL-10 (produced by MSCs) failed to upregulate maturation markers [31]. These immature DCs are strongly hampered in their ability to produce TNF-*α* and to promote inflammation [32].

In line with these observations, downregulated serum levels of TNF-*α* and increased serum concentration of IL-10, noticed in cisplatin + MSC-treated mice (Figure 1(d)), were accompanied with significantly lower number of renal-infiltrated TNF-*α*-producing DCs (Figure 2(c)).

MSCs are also able to suppress migration of DCs to the draining lymph nodes and inflamed tissues significantly affecting their ability for antigen presentation to CD4+ T helper (Th) cells and cross-presentation to CD8+ T cells [33, 34] resulting with attenuated generation and activation of IFN-*γ*-producing Th1 and IL-17-producing Th17 cells and CTLs [35]. Accordingly, we noticed reduced renal infiltration of IFN-*γ*- and IL-17-producing Th1 and Th17 cells (Figure 2(d)) and CTLs (Figure 2(e)), accompanied with decreased serum levels of IL-17 (Figure 1(d)) in cisplatin + MSC-treated mice when compared to cisplatin-only-treated animals. An important proinflammatory role of IL-17 in the nephrotoxicity induced by cisplatin was demonstrated by observing protection from cisplatin-induced functional and histological renal injury in IL-17 and ROR*γ*t-deficient mice and in mice treated with anti-IL-17 antibodies [36].

Homing of MSCs to the sites of renal injury and their integration and differentiation into tubular cells were rare or absent in animal models of acute kidney injury [37]. Accordingly, it is generally considered that MSCs exert their beneficial effects in paracrine manner, through the production of growth factors and cytokines that suppress oxidative stress, apoptosis, and inflammation in damaged kidneys [22, 23, 25, 38, 39]. In line with these findings, we showed here that MSC-CM also attenuates cisplatin-induced nephrotoxicity (Figure 3) in a similar manner as it was observed after injection of MSCs (Figure 1).

IDO and NO are important for MSC-mediated suppression of immune response in acute inflammation [25, 40]. IDO promotes the degradation of tryptophan into kynurenine and toxic metabolites (quinolinic acid and 3-hydroxy-anthranillic acid) which suppress proliferation or induce apoptosis of T cells. NO inhibits phosphorylation of signal transducer and activator of transcription-5 (Stat5) in T cells, leading to cell-cycle arrest [22, 23, 25]. Under inflammatory conditions, mouse MSCs increase expression of iNOS [38]. Activation of MSCs by TNF-*α* resulted with the increased expression of both iNOS and IDO (Figure 6(a)) whose interplay is important for MSC-mediated immunosuppression [40]. Since, in the presence of inflammatory cytokines, NO increased IDO activity [39], we assume that cisplatin-induced inflammation increased production of TNF-*α* in DCs (Figure 2(c)) resulting with increased generation and activation of IFN-*γ*-producing CD4+ Th1 cells and CD8+ CTLs (Figures 2(d) and 2(e)) that provoked MSCs to express iNOS and produce NO. MSC-derived NO increased IDO activity and MSC-mediated immunosuppression and led to the attenuation of cisplatin-induced cytotoxicity and inflammation (Figure 6(c)). In line with these observations are increased serum levels of immunosuppressive NO, kynurenine (product of IDO activity), and IL-10 (Figures 3(d), 3(e), and 3(f)) accompanied with reduced infiltration of immune cells in the kidneys of cisplatin-treated mice that received MSC-CM (Figure 3(h)) and attenuated concentration of kynurenine in supernatants of TNF-*α*-stimulated MSCs that were cultured in the presence of iNOS inhibitor, L-NMMA (Figure 6(b)). Blockade of iNOS by L-NMMA resulted with increased infiltration and activation of inflammatory DCs, effector T helper cells, and CTLs, decreased influx of tolerogenic DCs and regulatory T cells, and almost completely diminished renoprotective effects of MSC-CM (Figure 5).

In conclusion, our study provides the evidence that MSCs, in paracrine, iNOS-dependent manner, attenuate inflammation in cisplatin-induced nephrotoxicity by reducing influx and the capacity of immune cells, particularly DCs and T lymphocytes, to produce inflammatory cytokines. These findings could be helpful in developing new, MSC-based therapeutic approaches for attenuation of cisplatin-induced nephrotoxicity.
